# 2-Nitro-*N*-(4-pyridinio)benzene­sulfonamidate monohydrate

**DOI:** 10.1107/S1600536808035526

**Published:** 2008-11-08

**Authors:** Liang-Bin Hu, Jian Chen, Chang-Zhong Liu

**Affiliations:** a School of Food Science, Henan Institute of Science and Technology, Xinxiang 453003, People’s Republic of China; b College of Life Sciences, Nanjing Agricultural University, Nanjing 210095, People’s Republic of China; c College of Animal Science, Henan Institute of Science and Technology, Xinxiang 453003, People’s Republic of China

## Abstract

The title compound, C_11_H_9_N_3_O_4_S·H_2_O, contains both an acid and a base centre and displays a zwitterionic structure. There are two independent mol­ecules and two water mol­ecules in the asymmetric unit. The dihedral angles between the benzene ring and the pyridinium ring are 109.7 (1) and 110.7 (1)°. The dihedral angles between the nitro group and the benzene ring are 116.1 (2) and 116.7 (1)°. The crystal structure is stabilized by N—H⋯O, O—H⋯N and O—H⋯O hydrogen bonds.

## Related literature

For the uses of organic pyridinium salts, see: Damiano *et al.* (2007 ▶). For zwitterionic forms of *N*-aryl­benzene­sulfonamides, see: Li *et al.* (2007 ▶); Yu & Li (2007 ▶). For reference geometric data, see: Allen *et al.* (1987 ▶).
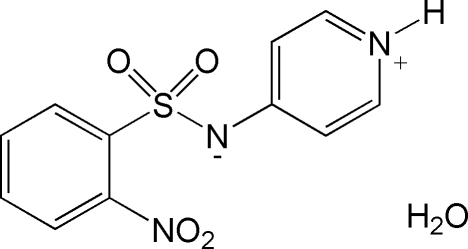


## Experimental

### 

#### Crystal data

C_11_H_9_N_3_O_4_S·H_2_O
*M* *_r_* = 297.29
Monoclinic, 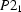

*a* = 8.7206 (17) Å
*b* = 11.972 (2) Å
*c* = 12.743 (3) Å
β = 107.65 (3)°
*V* = 1267.8 (4) Å^3^
*Z* = 4
Mo *K*α radiation
μ = 0.28 mm^−1^
*T* = 113 (2) K
0.14 × 0.10 × 0.04 mm

#### Data collection

Rigaku Saturn diffractometer
Absorption correction: multi-scan (*CrystalClear*; Rigaku/MSC, 2005 ▶) *T*_min_ = 0.962, *T*_max_ = 0.989
10348 measured reflections
4633 independent reflections
3587 reflections with *I* > 2σ(*I*)
*R*_int_ = 0.033

#### Refinement

*R*[*F* ^2^ > 2σ(*F* ^2^)] = 0.032
*wR*(*F* ^2^) = 0.090
*S* = 1.03
4633 reflections
385 parameters
9 restraints
H atoms treated by a mixture of independent and constrained refinement
Δρ_max_ = 0.22 e Å^−3^
Δρ_min_ = −0.40 e Å^−3^
Absolute structure: Flack (1983 ▶), 1560 Friedel pairs
Flack parameter: 0.52 (6)

### 

Data collection: *CrystalClear* (Rigaku/MSC, 2005 ▶); cell refinement: *CrystalClear*; data reduction: *CrystalClear*; program(s) used to solve structure: *SHELXS97* (Sheldrick, 2008 ▶); program(s) used to refine structure: *SHELXL97* (Sheldrick, 2008 ▶); molecular graphics: *SHELXTL* (Sheldrick, 2008 ▶); software used to prepare material for publication: *SHELXTL*.

## Supplementary Material

Crystal structure: contains datablocks global, I. DOI: 10.1107/S1600536808035526/hg2431sup1.cif

Structure factors: contains datablocks I. DOI: 10.1107/S1600536808035526/hg2431Isup2.hkl

Additional supplementary materials:  crystallographic information; 3D view; checkCIF report

## Figures and Tables

**Table 1:** Hydrogen-bond geometry (Å, °)

*D*—H⋯*A*	*D*—H	H⋯*A*	*D*⋯*A*	*D*—H⋯*A*
N4—H4*A*⋯O9	0.913 (10)	1.868 (12)	2.764 (3)	167 (3)
O9—H9*A*⋯O1	0.864 (10)	2.122 (11)	2.967 (3)	166 (3)
N1—H1*A*⋯O10^i^	0.918 (10)	1.843 (11)	2.757 (3)	174 (4)
O9—H9*B*⋯N5^ii^	0.867 (10)	2.043 (11)	2.901 (3)	170 (2)
O9—H9*B*⋯O6^ii^	0.867 (10)	2.56 (2)	3.125 (3)	123 (2)
O10—H10*A*⋯O6^iii^	0.867 (10)	2.051 (11)	2.914 (3)	174 (4)
O10—H10*B*⋯N2^iv^	0.870 (10)	2.042 (12)	2.902 (3)	170 (3)

Symmetry codes: (i) 
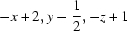
; (ii) 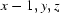; (iii) 
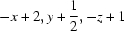
; (iv) 
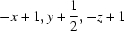
.

**Fig. 1.**
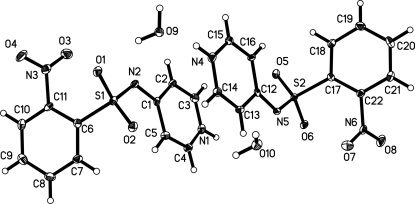
View of the molecule of title compound showing the atom-labelling scheme. Displacement ellipsoids are drawn at the 35% probability level (arbitrary spheres for the H atoms).

## supplementary crystallographic information

### Comment

Organic pyridinium salts have been widely used in the construction of supramolecular architectures (Damiano *et al.*, 2007). As part of our ongoing studies of supramolecular chemistry involving the pyridinium rings (Li *et al.*, 2007), the structure of the title compound was determined by X-ray diffraction. There are two independent molecules and two independent water molecules in the unit cell. In the cations of the title compound the short C—N distance [N2—C1 = 1.367 (3) Å and N5—C12 = 1.365 (3) Å] indicate the slight conjugation of the sulfonamide N with the pyridinium ring (Allen *et al.*, 1987).

The dihedral angles between the benzene ring and the pyridinium ring are 109.7 (1)° and 110.7 (1)° respectively. And the dihedral angles between the nitro group and the benzene ring are 116.1 (2)° and 116.7 (1)° respectively. The crystal structure is stabilized by N—H···O hydrogen bonds.

### Experimental

A solution of 2-nitrobenzenesulfonyl chloride (2.2 g, 10 mmol) in CH_2_Cl_2_ (10 ml) was added dropwise to a suspension of 4-aminopyridine (0.9 g, 10 mmol) in CH_2_Cl_2_ (10 ml) at room temperature with stirring. The reaction mixture was stirred overnight. The yellow solid obtained was washed with warm water to obtain the title compound in a yield of 55.7%. A colorless single crystal suitable for X-ray analysis was obtained by slow evaporation of an NaOH (10%) solution at room temperature over a period of a week.

### Refinement

The N-bound H atoms were located in a difference map and their coordinates were refined with *U*_iso_(H) = 1.2*U*_eq_(N). The C-bound H atoms were positioned geometrically (C—H = 0.93 Å) and refined as riding with *U*_iso_(H) = 1.2*U*_eq_(C). The Flack test results are ambiguous because of the presence of merohedral twin. The water O-bound H atoms were refined freely, but the O—H distances were restrained to 0.85 (1) Å, and the water HA···HB distance to 1.45 (1) Å.

### Crystal data

**Table:**

C_11_H_9_N_3_O_4_S·H_2_O	*F*_000_ = 616
*M**_r_* = 297.29	*D*_x_ = 1.557 Mg m^−^^3^
Monoclinic, *P*2_1_	Mo *K*α radiation λ = 0.71073 Å
Hall symbol: P 2yb	Cell parameters from 4439 reflections
*a* = 8.7206 (17) Å	θ = 2.4–27.5º
*b* = 11.972 (2) Å	µ = 0.28 mm^−^^1^
*c* = 12.743 (3) Å	*T* = 113 (2) K
β = 107.65 (3)º	Block, colourless
*V* = 1267.8 (4) Å^3^	0.14 × 0.10 × 0.04 mm
*Z* = 4	

### Data collection

**Table:**

Rigaku Saturn diffractometer	4633 independent reflections
Radiation source: rotating anode	3587 reflections with *I* > 2σ(*I*)
Monochromator: confocal	*R*_int_ = 0.033
*T* = 113(2) K	θ_max_ = 27.5º
ω scans	θ_min_ = 1.7º
Absorption correction: multi-scan(CrystalClear; Rigaku/MSC, 2005)	*h* = −11→11
*T*_min_ = 0.962, *T*_max_ = 0.989	*k* = −12→15
10348 measured reflections	*l* = −16→16

### Refinement

**Table:**

Refinement on *F*^2^	Hydrogen site location: inferred from neighbouring sites
Least-squares matrix: full	H atoms treated by a mixture of independent and constrained refinement
*R*[*F*^2^ > 2σ(*F*^2^)] = 0.032	*w* = 1/[σ^2^(*F*_o_^2^) + (0.0539*P*)^2^] where *P* = (*F*_o_^2^ + 2*F*_c_^2^)/3
*wR*(*F*^2^) = 0.090	(Δ/σ)_max_ = 0.002
*S* = 1.03	Δρ_max_ = 0.22 e Å^−^^3^
4633 reflections	Δρ_min_ = −0.40 e Å^−^^3^
385 parameters	Extinction correction: none
9 restraints	Absolute structure: Flack (1983), with how many Friedel pairs?
Primary atom site location: structure-invariant direct methods	Flack parameter: 0.52 (6)
Secondary atom site location: difference Fourier map	

### Special details

**Table:**

Geometry. All e.s.d.'s (except the e.s.d. in the dihedral angle between two l.s. planes) are estimated using the full covariance matrix. The cell e.s.d.'s are taken into account individually in the estimation of e.s.d.'s in distances, angles and torsion angles; correlations between e.s.d.'s in cell parameters are only used when they are defined by crystal symmetry. An approximate (isotropic) treatment of cell e.s.d.'s is used for estimating e.s.d.'s involving l.s. planes.
Refinement. Refinement of *F*^2^ against ALL reflections. The weighted *R*-factor *wR* and goodness of fit *S* are based on *F*^2^, conventional *R*-factors *R* are based on *F*, with *F* set to zero for negative *F*^2^. The threshold expression of *F*^2^ > σ(*F*^2^) is used only for calculating *R*-factors(gt) *etc*. and is not relevant to the choice of reflections for refinement. *R*-factors based on *F*^2^ are statistically about twice as large as those based on *F*, and *R*- factors based on ALL data will be even larger.

### Fractional atomic coordinates and isotropic or equivalent isotropic displacement parameters (Å^2^)

**Table:**

	*x*	*y*	*z*	*U*_iso_*/*U*_eq_	
S1	0.54299 (7)	0.49945 (5)	0.75574 (5)	0.01423 (14)	
S2	1.01577 (7)	0.25356 (5)	0.35546 (5)	0.01307 (14)	
O1	0.36887 (19)	0.49815 (17)	0.72910 (14)	0.0177 (4)	
O2	0.6116 (2)	0.59540 (15)	0.71745 (16)	0.0187 (4)	
O3	0.4219 (3)	0.29003 (18)	0.87441 (18)	0.0318 (5)	
O4	0.2777 (2)	0.4008 (2)	0.94108 (18)	0.0326 (5)	
O5	0.9524 (2)	0.15286 (15)	0.38857 (15)	0.0165 (4)	
O6	1.1904 (2)	0.26159 (18)	0.38501 (16)	0.0182 (4)	
O7	1.1373 (3)	0.46983 (18)	0.24125 (19)	0.0334 (5)	
O8	1.2807 (2)	0.3638 (2)	0.16880 (18)	0.0371 (6)	
N1	1.0324 (3)	0.2999 (2)	0.67719 (19)	0.0185 (5)	
N2	0.5920 (2)	0.38299 (18)	0.71702 (19)	0.0146 (5)	
N3	0.4050 (3)	0.3727 (2)	0.9245 (2)	0.0217 (5)	
N4	0.5077 (3)	0.4240 (2)	0.43389 (18)	0.0179 (5)	
N5	0.9564 (2)	0.36491 (18)	0.39696 (18)	0.0141 (5)	
N6	1.1564 (3)	0.3888 (2)	0.18846 (19)	0.0230 (6)	
C1	0.7414 (3)	0.3615 (2)	0.7078 (2)	0.0132 (5)	
C2	0.7644 (3)	0.2549 (2)	0.6690 (2)	0.0167 (5)	
H2	0.6813	0.2029	0.6537	0.020*	
C3	0.9100 (3)	0.2265 (2)	0.6532 (2)	0.0188 (6)	
H3	0.9231	0.1563	0.6259	0.023*	
C4	1.0168 (3)	0.4019 (2)	0.7153 (2)	0.0179 (6)	
H4	1.1030	0.4513	0.7304	0.021*	
C5	0.8752 (3)	0.4350 (2)	0.7325 (2)	0.0169 (6)	
H5	0.8672	0.5058	0.7604	0.020*	
C6	0.6158 (3)	0.5053 (3)	0.9027 (2)	0.0148 (5)	
C7	0.7441 (3)	0.5753 (2)	0.9531 (2)	0.0189 (6)	
H7	0.7936	0.6156	0.9100	0.023*	
C8	0.7996 (3)	0.5858 (2)	1.0674 (2)	0.0240 (6)	
H8	0.8857	0.6329	1.0999	0.029*	
C9	0.7271 (3)	0.5264 (3)	1.1326 (2)	0.0245 (7)	
H9	0.7656	0.5325	1.2089	0.029*	
C10	0.5978 (3)	0.4582 (2)	1.0843 (2)	0.0208 (6)	
H10	0.5469	0.4197	1.1277	0.025*	
C11	0.5441 (3)	0.4472 (2)	0.9712 (2)	0.0162 (6)	
C12	0.8049 (3)	0.3782 (2)	0.4049 (2)	0.0120 (5)	
C13	0.7734 (3)	0.4806 (2)	0.4510 (2)	0.0163 (6)	
H13	0.8534	0.5347	0.4722	0.020*	
C14	0.6264 (3)	0.4998 (2)	0.4642 (2)	0.0181 (5)	
H14	0.6078	0.5669	0.4949	0.022*	
C15	0.5302 (3)	0.3265 (2)	0.3884 (2)	0.0165 (5)	
H15	0.4464	0.2752	0.3672	0.020*	
C16	0.6767 (3)	0.3014 (2)	0.3725 (2)	0.0136 (5)	
H16	0.6905	0.2339	0.3405	0.016*	
C17	0.9459 (3)	0.2526 (2)	0.2084 (2)	0.0145 (5)	
C18	0.8167 (3)	0.1850 (2)	0.1552 (2)	0.0179 (6)	
H18	0.7672	0.1423	0.1967	0.021*	
C19	0.7603 (3)	0.1799 (2)	0.0417 (2)	0.0229 (6)	
H19	0.6723	0.1349	0.0080	0.027*	
C20	0.8324 (3)	0.2406 (3)	−0.0223 (2)	0.0245 (7)	
H20	0.7933	0.2365	−0.0987	0.029*	
C21	0.9631 (3)	0.3074 (2)	0.0276 (2)	0.0195 (6)	
H21	1.0141	0.3476	−0.0146	0.023*	
C22	1.0173 (3)	0.3137 (2)	0.1415 (2)	0.0157 (6)	
O9	0.2408 (2)	0.50194 (18)	0.48520 (16)	0.0183 (4)	
O10	0.6923 (2)	0.74683 (18)	0.37557 (18)	0.0203 (4)	
H1A	1.127 (3)	0.280 (3)	0.665 (3)	0.054 (11)*	
H4A	0.411 (2)	0.441 (2)	0.444 (2)	0.021 (8)*	
H9A	0.260 (3)	0.500 (3)	0.5558 (8)	0.047 (11)*	
H9B	0.160 (2)	0.460 (2)	0.4517 (18)	0.024 (9)*	
H10A	0.719 (4)	0.751 (4)	0.4468 (8)	0.11 (2)*	
H10B	0.601 (2)	0.780 (3)	0.346 (2)	0.034 (10)*	

### Atomic displacement parameters (Å^2^)

**Table:**

	*U*^11^	*U*^22^	*U*^33^	*U*^12^	*U*^13^	*U*^23^
S1	0.0127 (3)	0.0152 (3)	0.0156 (3)	0.0016 (3)	0.0055 (2)	0.0026 (3)
S2	0.0098 (3)	0.0162 (3)	0.0138 (3)	0.0013 (3)	0.0044 (2)	0.0012 (3)
O1	0.0119 (8)	0.0220 (10)	0.0201 (10)	0.0049 (9)	0.0064 (7)	0.0009 (9)
O2	0.0242 (10)	0.0133 (9)	0.0209 (11)	0.0000 (8)	0.0102 (9)	0.0042 (8)
O3	0.0376 (12)	0.0285 (12)	0.0288 (13)	−0.0115 (10)	0.0095 (10)	−0.0019 (10)
O4	0.0157 (9)	0.0515 (15)	0.0329 (13)	−0.0051 (10)	0.0109 (9)	0.0064 (12)
O5	0.0194 (10)	0.0153 (9)	0.0158 (10)	0.0043 (8)	0.0067 (8)	0.0053 (8)
O6	0.0099 (8)	0.0269 (11)	0.0190 (10)	0.0008 (9)	0.0061 (7)	0.0016 (9)
O7	0.0378 (13)	0.0292 (13)	0.0329 (13)	−0.0111 (10)	0.0101 (11)	−0.0060 (10)
O8	0.0180 (10)	0.0689 (18)	0.0274 (13)	−0.0077 (11)	0.0114 (10)	0.0055 (12)
N1	0.0135 (11)	0.0249 (12)	0.0184 (12)	0.0064 (10)	0.0067 (9)	0.0022 (10)
N2	0.0116 (10)	0.0147 (11)	0.0196 (12)	0.0000 (9)	0.0078 (9)	−0.0001 (9)
N3	0.0209 (12)	0.0263 (14)	0.0176 (13)	−0.0040 (11)	0.0057 (10)	0.0071 (10)
N4	0.0148 (11)	0.0224 (13)	0.0179 (12)	0.0020 (10)	0.0072 (9)	0.0009 (10)
N5	0.0108 (10)	0.0177 (11)	0.0144 (11)	−0.0028 (9)	0.0046 (9)	−0.0035 (9)
N6	0.0193 (12)	0.0354 (15)	0.0146 (12)	−0.0067 (11)	0.0054 (10)	0.0064 (11)
C1	0.0123 (11)	0.0178 (13)	0.0100 (13)	0.0024 (10)	0.0042 (10)	0.0041 (10)
C2	0.0166 (11)	0.0179 (12)	0.0164 (13)	−0.0044 (12)	0.0062 (10)	0.0015 (12)
C3	0.0177 (13)	0.0173 (14)	0.0217 (15)	0.0033 (11)	0.0062 (11)	−0.0025 (11)
C4	0.0133 (12)	0.0214 (14)	0.0181 (14)	−0.0009 (11)	0.0035 (10)	0.0014 (12)
C5	0.0145 (12)	0.0147 (13)	0.0211 (15)	0.0009 (11)	0.0048 (11)	−0.0006 (11)
C6	0.0151 (12)	0.0134 (13)	0.0166 (13)	0.0032 (12)	0.0059 (10)	0.0000 (12)
C7	0.0206 (13)	0.0143 (13)	0.0224 (15)	−0.0036 (11)	0.0075 (12)	0.0006 (11)
C8	0.0252 (15)	0.0225 (15)	0.0224 (16)	−0.0067 (13)	0.0040 (13)	−0.0035 (12)
C9	0.0255 (14)	0.0278 (16)	0.0184 (15)	0.0041 (13)	0.0038 (12)	−0.0030 (12)
C10	0.0236 (14)	0.0239 (15)	0.0178 (15)	0.0041 (12)	0.0106 (12)	0.0047 (12)
C11	0.0103 (11)	0.0175 (13)	0.0217 (15)	0.0013 (10)	0.0062 (11)	0.0027 (11)
C12	0.0152 (12)	0.0125 (12)	0.0083 (12)	0.0024 (10)	0.0038 (10)	0.0017 (10)
C13	0.0168 (12)	0.0141 (13)	0.0183 (14)	−0.0048 (11)	0.0060 (11)	−0.0026 (11)
C14	0.0225 (13)	0.0147 (12)	0.0186 (13)	−0.0018 (12)	0.0087 (11)	−0.0020 (12)
C15	0.0136 (12)	0.0198 (13)	0.0173 (14)	0.0002 (11)	0.0066 (11)	−0.0031 (11)
C16	0.0093 (11)	0.0150 (12)	0.0173 (14)	−0.0026 (10)	0.0051 (10)	−0.0032 (11)
C17	0.0160 (12)	0.0150 (12)	0.0119 (13)	0.0007 (12)	0.0033 (10)	−0.0004 (11)
C18	0.0193 (13)	0.0163 (14)	0.0198 (15)	−0.0015 (12)	0.0084 (12)	0.0037 (12)
C19	0.0262 (15)	0.0190 (15)	0.0202 (15)	−0.0061 (13)	0.0022 (12)	0.0005 (12)
C20	0.0322 (15)	0.0275 (16)	0.0115 (14)	0.0032 (14)	0.0033 (12)	−0.0006 (13)
C21	0.0193 (13)	0.0221 (15)	0.0205 (15)	0.0031 (12)	0.0110 (12)	0.0049 (12)
C22	0.0118 (11)	0.0181 (14)	0.0172 (14)	0.0001 (11)	0.0045 (11)	0.0019 (11)
O9	0.0157 (9)	0.0210 (10)	0.0194 (11)	0.0003 (9)	0.0072 (8)	−0.0056 (10)
O10	0.0170 (9)	0.0221 (10)	0.0215 (11)	0.0022 (9)	0.0057 (8)	0.0043 (10)

### Geometric parameters (Å, °)

**Table:**

S1—O2	1.4464 (19)	C6—C11	1.403 (3)
S1—O1	1.4521 (17)	C7—C8	1.394 (4)
S1—N2	1.581 (2)	C7—H7	0.9300
S1—C6	1.787 (3)	C8—C9	1.383 (4)
S2—O5	1.4416 (19)	C8—H8	0.9300
S2—O6	1.4568 (17)	C9—C10	1.377 (4)
S2—N5	1.578 (2)	C9—H9	0.9300
S2—C17	1.787 (3)	C10—C11	1.380 (4)
O3—N3	1.211 (3)	C10—H10	0.9300
O4—N3	1.237 (3)	C12—C16	1.409 (3)
O7—N6	1.221 (3)	C12—C13	1.421 (3)
O8—N6	1.221 (3)	C13—C14	1.362 (3)
N1—C4	1.336 (4)	C13—H13	0.9300
N1—C3	1.345 (3)	C14—H14	0.9300
N1—H1A	0.918 (10)	C15—C16	1.385 (3)
N2—C1	1.367 (3)	C15—H15	0.9300
N3—C11	1.478 (3)	C16—H16	0.9300
N4—C14	1.342 (3)	C17—C18	1.386 (4)
N4—C15	1.344 (3)	C17—C22	1.404 (3)
N4—H4A	0.913 (10)	C18—C19	1.380 (4)
N5—C12	1.365 (3)	C18—H18	0.9300
N6—C22	1.482 (3)	C19—C20	1.379 (4)
C1—C2	1.405 (4)	C19—H19	0.9300
C1—C5	1.419 (4)	C20—C21	1.380 (4)
C2—C3	1.387 (3)	C20—H20	0.9300
C2—H2	0.9300	C21—C22	1.385 (4)
C3—H3	0.9300	C21—H21	0.9300
C4—C5	1.376 (3)	O9—H9A	0.864 (10)
C4—H4	0.9300	O9—H9B	0.867 (10)
C5—H5	0.9300	O10—H10A	0.867 (10)
C6—C7	1.389 (4)	O10—H10B	0.870 (10)
			
O2—S1—O1	116.08 (11)	C9—C8—C7	120.3 (3)
O2—S1—N2	114.62 (10)	C9—C8—H8	119.9
O1—S1—N2	106.27 (11)	C7—C8—H8	119.9
O2—S1—C6	105.68 (13)	C10—C9—C8	119.8 (3)
O1—S1—C6	105.03 (11)	C10—C9—H9	120.1
N2—S1—C6	108.55 (13)	C8—C9—H9	120.1
O5—S2—O6	116.29 (11)	C9—C10—C11	119.8 (3)
O5—S2—N5	114.48 (10)	C9—C10—H10	120.1
O6—S2—N5	106.33 (12)	C11—C10—H10	120.1
O5—S2—C17	105.23 (12)	C10—C11—C6	121.9 (3)
O6—S2—C17	105.61 (11)	C10—C11—N3	117.1 (2)
N5—S2—C17	108.33 (12)	C6—C11—N3	121.0 (2)
C4—N1—C3	121.3 (2)	N5—C12—C16	127.0 (2)
C4—N1—H1A	120 (2)	N5—C12—C13	116.4 (2)
C3—N1—H1A	119 (2)	C16—C12—C13	116.6 (2)
C1—N2—S1	123.21 (19)	C14—C13—C12	120.2 (2)
O3—N3—O4	124.9 (3)	C14—C13—H13	119.9
O3—N3—C11	119.3 (2)	C12—C13—H13	119.9
O4—N3—C11	115.9 (2)	N4—C14—C13	121.6 (3)
C14—N4—C15	120.7 (2)	N4—C14—H14	119.2
C14—N4—H4A	118.3 (19)	C13—C14—H14	119.2
C15—N4—H4A	121.0 (19)	N4—C15—C16	120.9 (2)
C12—N5—S2	123.16 (18)	N4—C15—H15	119.6
O7—N6—O8	125.8 (3)	C16—C15—H15	119.6
O7—N6—C22	118.2 (2)	C15—C16—C12	120.0 (2)
O8—N6—C22	115.9 (2)	C15—C16—H16	120.0
N2—C1—C2	116.3 (2)	C12—C16—H16	120.0
N2—C1—C5	127.2 (2)	C18—C17—C22	116.9 (2)
C2—C1—C5	116.5 (2)	C18—C17—S2	119.0 (2)
C3—C2—C1	120.6 (2)	C22—C17—S2	124.1 (2)
C3—C2—H2	119.7	C19—C18—C17	121.1 (2)
C1—C2—H2	119.7	C19—C18—H18	119.4
N1—C3—C2	120.3 (2)	C17—C18—H18	119.4
N1—C3—H3	119.8	C20—C19—C18	121.0 (3)
C2—C3—H3	119.8	C20—C19—H19	119.5
N1—C4—C5	121.1 (3)	C18—C19—H19	119.5
N1—C4—H4	119.5	C19—C20—C21	119.6 (3)
C5—C4—H4	119.5	C19—C20—H20	120.2
C4—C5—C1	120.2 (2)	C21—C20—H20	120.2
C4—C5—H5	119.9	C20—C21—C22	119.1 (3)
C1—C5—H5	119.9	C20—C21—H21	120.4
C7—C6—C11	117.3 (2)	C22—C21—H21	120.4
C7—C6—S1	119.3 (2)	C21—C22—C17	122.3 (2)
C11—C6—S1	123.3 (2)	C21—C22—N6	115.7 (2)
C6—C7—C8	120.9 (3)	C17—C22—N6	122.0 (2)
C6—C7—H7	119.6	H9A—O9—H9B	111.6 (16)
C8—C7—H7	119.6	H10A—O10—H10B	110.7 (16)
			
O2—S1—N2—C1	36.7 (2)	O4—N3—C11—C10	62.9 (3)
O1—S1—N2—C1	166.3 (2)	O3—N3—C11—C6	64.4 (4)
C6—S1—N2—C1	−81.2 (2)	O4—N3—C11—C6	−116.0 (3)
O5—S2—N5—C12	−36.0 (2)	S2—N5—C12—C16	−4.2 (4)
O6—S2—N5—C12	−165.8 (2)	S2—N5—C12—C13	175.7 (2)
C17—S2—N5—C12	81.0 (2)	N5—C12—C13—C14	−178.5 (2)
S1—N2—C1—C2	−178.07 (19)	C16—C12—C13—C14	1.4 (4)
S1—N2—C1—C5	1.5 (4)	C15—N4—C14—C13	−0.6 (4)
N2—C1—C2—C3	177.8 (2)	C12—C13—C14—N4	−0.4 (4)
C5—C1—C2—C3	−1.9 (4)	C14—N4—C15—C16	0.6 (4)
C4—N1—C3—C2	−0.8 (4)	N4—C15—C16—C12	0.4 (4)
C1—C2—C3—N1	1.4 (4)	N5—C12—C16—C15	178.5 (2)
C3—N1—C4—C5	0.7 (4)	C13—C12—C16—C15	−1.3 (4)
N1—C4—C5—C1	−1.2 (4)	O5—S2—C17—C18	18.6 (2)
N2—C1—C5—C4	−177.8 (3)	O6—S2—C17—C18	142.2 (2)
C2—C1—C5—C4	1.8 (4)	N5—S2—C17—C18	−104.2 (2)
O2—S1—C6—C7	−14.8 (2)	O5—S2—C17—C22	−159.0 (2)
O1—S1—C6—C7	−138.0 (2)	O6—S2—C17—C22	−35.5 (3)
N2—S1—C6—C7	108.6 (2)	N5—S2—C17—C22	78.1 (2)
O2—S1—C6—C11	161.6 (2)	C22—C17—C18—C19	−1.0 (4)
O1—S1—C6—C11	38.3 (3)	S2—C17—C18—C19	−178.8 (2)
N2—S1—C6—C11	−75.0 (2)	C17—C18—C19—C20	1.1 (4)
C11—C6—C7—C8	0.6 (4)	C18—C19—C20—C21	0.1 (4)
S1—C6—C7—C8	177.2 (2)	C19—C20—C21—C22	−1.2 (4)
C6—C7—C8—C9	−0.1 (4)	C20—C21—C22—C17	1.3 (4)
C7—C8—C9—C10	−1.1 (4)	C20—C21—C22—N6	−179.0 (2)
C8—C9—C10—C11	1.7 (4)	C18—C17—C22—C21	−0.2 (4)
C9—C10—C11—C6	−1.2 (4)	S2—C17—C22—C21	177.5 (2)
C9—C10—C11—N3	180.0 (2)	C18—C17—C22—N6	−179.9 (2)
C7—C6—C11—C10	0.0 (4)	S2—C17—C22—N6	−2.2 (4)
S1—C6—C11—C10	−176.4 (2)	O7—N6—C22—C21	116.6 (3)
C7—C6—C11—N3	178.8 (2)	O8—N6—C22—C21	−62.4 (3)
S1—C6—C11—N3	2.4 (4)	O7—N6—C22—C17	−63.7 (4)
O3—N3—C11—C10	−116.7 (3)	O8—N6—C22—C17	117.3 (3)

### Hydrogen-bond geometry (Å, °)

**Table:**

*D*—H···*A*	*D*—H	H···*A*	*D*···*A*	*D*—H···*A*
N4—H4A···O9	0.913 (10)	1.868 (12)	2.764 (3)	167 (3)
O9—H9A···O1	0.864 (10)	2.122 (11)	2.967 (3)	166 (3)
N1—H1A···O10^i^	0.918 (10)	1.843 (11)	2.757 (3)	174 (4)
O9—H9B···N5^ii^	0.867 (10)	2.043 (11)	2.901 (3)	170 (2)
O9—H9B···O6^ii^	0.867 (10)	2.56 (2)	3.125 (3)	123 (2)
O10—H10A···O6^iii^	0.867 (10)	2.051 (11)	2.914 (3)	174 (4)
O10—H10B···N2^iv^	0.870 (10)	2.042 (12)	2.902 (3)	170 (3)

Symmetry codes: (i) −*x*+2, *y*−1/2, −*z*+1; (ii) *x*−1, *y*, *z*; (iii) −*x*+2, *y*+1/2, −*z*+1; (iv) −*x*+1, *y*+1/2, −*z*+1.
